# Ipriflavone attenuates the degeneration of cartilage by blocking the Indian hedgehog pathway

**DOI:** 10.1186/s13075-019-1895-x

**Published:** 2019-05-02

**Authors:** Li Guo, Xiaochun Wei, Zhiwei Zhang, Xiaojian Wang, Chunli Wang, Pengcui Li, Chunfang Wang, Lei Wei

**Affiliations:** 1 Department of Orthopedics, Second Hospital of Shanxi Medical University, Shanxi Key Laboratory of Bone and Soft Tissue Injury Repair, Taiyuan, China No. 382, Wuyi Road, Taiyuan, 030001 China; 2 Shanxi Key Laboratory of Laboratory Animal and Animal Model of Human Diseases, Department of Experimental Animal Center, Shanxi Medical University, No. 56, Xinjian Southern Road, Taiyuan, 030001 China; 3 Department of Orthopedics, Warren Alpert Medical School of Brown University, Suite 402A, 1 Hoppin Street, Providence, RI 02903 USA

**Keywords:** Ipriflavone, Osteoarthritis, Indian hedgehog, Chondrocyte hypertrophy

## Abstract

**Background:**

To determine if ipriflavone, a novel and safe inhibitor of Indian hedgehog (Ihh) signaling, can attenuate cartilage degeneration by blocking the Ihh pathway.

**Methods:**

Human chondrocytes were used to evaluate Ihh signaling, cell proliferation, apoptosis, gene, and protein expression of chondrocytes by cell proliferation and apoptosis assays, real-time qPCR, and Western blotting at 48 h after ipriflavone treatment. Human cartilage explants were further used to validate the cell culture results. The effects of ipriflavone on cartilage degeneration in vivo were assessed using the rat ACLT OA model. Two-month-old male SD rats were randomized into 3 groups (*n* = 75): (1) sham, (2) ACLT alone, and (3) ACLT+ ipriflavone. Ipriflavone was administered intragastrically at 24 h after ACLT for 6 weeks. The extent of OA progression was evaluated by the OARSI score and immunohistochemistry at 12 weeks after surgery. The Ihh signaling pathway and OA-related genes were quantified by real-time PCR.

**Results:**

Cell proliferation in the cells treated with ipriflavone was increased to 36.40% ± 1.32% (5 μM) and 28.54% ± 0.74% (10 μM) from 11.99% ± 0.35% (DMSO) (*P* < 0.001), and apoptosis was decreased to 12.64% ± 3.7% (5 μM) and 15.18% ± 3.13% (10 μM) from 25.76% ± 5.1% (DMSO) (*P* < 0.05). Ipriflavone blocked Runx-2 mainly through the Smo-Gli2 pathway. A similar result was found in the cartilage explant culture. Ihh signaling in vivo was inhibited in animals treated with ipriflavone. Safranin-O staining revealed a less cartilage damage with lower OARSI scores (*P* < 0.05) in the ipriflavone-treated animals compared with untreated animals. The gene expression of Smo and Gli2 was inhibited significantly by ipriflavone (*P* < 0.05). The OA-related gene and protein type X, MMP-13, and type II collagen-C fragment were reduced, while type II collagen and Agg were increased in the ipriflavone-treated animals (*P* < 0.05).

**Conclusions:**

Catabolic genes were disrupted by blocking the Ihh pathway. This finding suggests that disruption of Ihh signaling with ipriflavone provides chondral protection in rat posttraumatic OA.

**Electronic supplementary material:**

The online version of this article (10.1186/s13075-019-1895-x) contains supplementary material, which is available to authorized users.

## Background

Osteoarthritis (OA) is one of the most common musculoskeletal diseases with aging. OA is characterized by progressive degeneration of the articular cartilage and joint swelling, pain, stiffness, and loss of mobility. These characteristics not only significantly affect the quality of life but also aggravate the financial burden of patients [1]. However, the underlying molecular mechanisms involved in the pathogenesis and progression of OA are still unknown, and no disease-modifying therapy is available.

Recently, we and others found a significant increase in Indian hedgehog (Ihh) protein expression in human osteoarthritic cartilage, which promotes chondrocyte hypertrophy and the upregulation of matrix metallopeptidase 13 (MMP-13) [2–7]. We further demonstrated that disrupting the Ihh signaling pathway in vivo attenuates surgically induced OA progression in Col2a1-CreERT2; Ihh fl/fl mice [8]. This evidence indicates that upregulation of the Ihh pathway plays a key role during OA progression and that inhibiting the Ihh pathway may attenuate the degeneration of cartilage. However, Ihh gene deletion is not a therapeutic option in humans, and most chemical inhibitors of hedgehog (Hh) signaling induce severe side effects, including holoprosencephaly, cleft palate, and limb defects [9–13]. Therefore, a safe inhibitor of the Hh pathway would be extremely important for human OA treatment.

Recently, three Hh pathway inhibitors have been identified among 4240 compounds using small molecule screening: (1) ipriflavone, a dietary supplement; (2) tolnaftate, an antifungal agent; (3) and 17-b-estradiol, a human hormone and pharmaceutical agent [14]. These compounds demonstrate similar efficiencies of Hh signaling inhibition in both mouse and human cells without cytotoxicity, and they are 8- to 30-fold safer than the index Hh pathway inhibitor cyclopamine. For this study, we choose ipriflavone, a synthetic isoflavone that is used worldwide as a supplement for its touted anabolic and bone density-building properties [15–19] to test our hypothesis: blockade of the Ihh pathway with ipriflavone alleviates the degeneration of cartilage in vitro and in vivo because it has been used to treat diseases, such as breast cancer and diabetes in rats via inhibiting the Ihh pathway [20, 21], and the pharmacokinetics of Ipriflavone have been described in animal models [22] and humans [23].

## Methods

### Chondrocyte isolation and primary culture

**Fig. 1:**
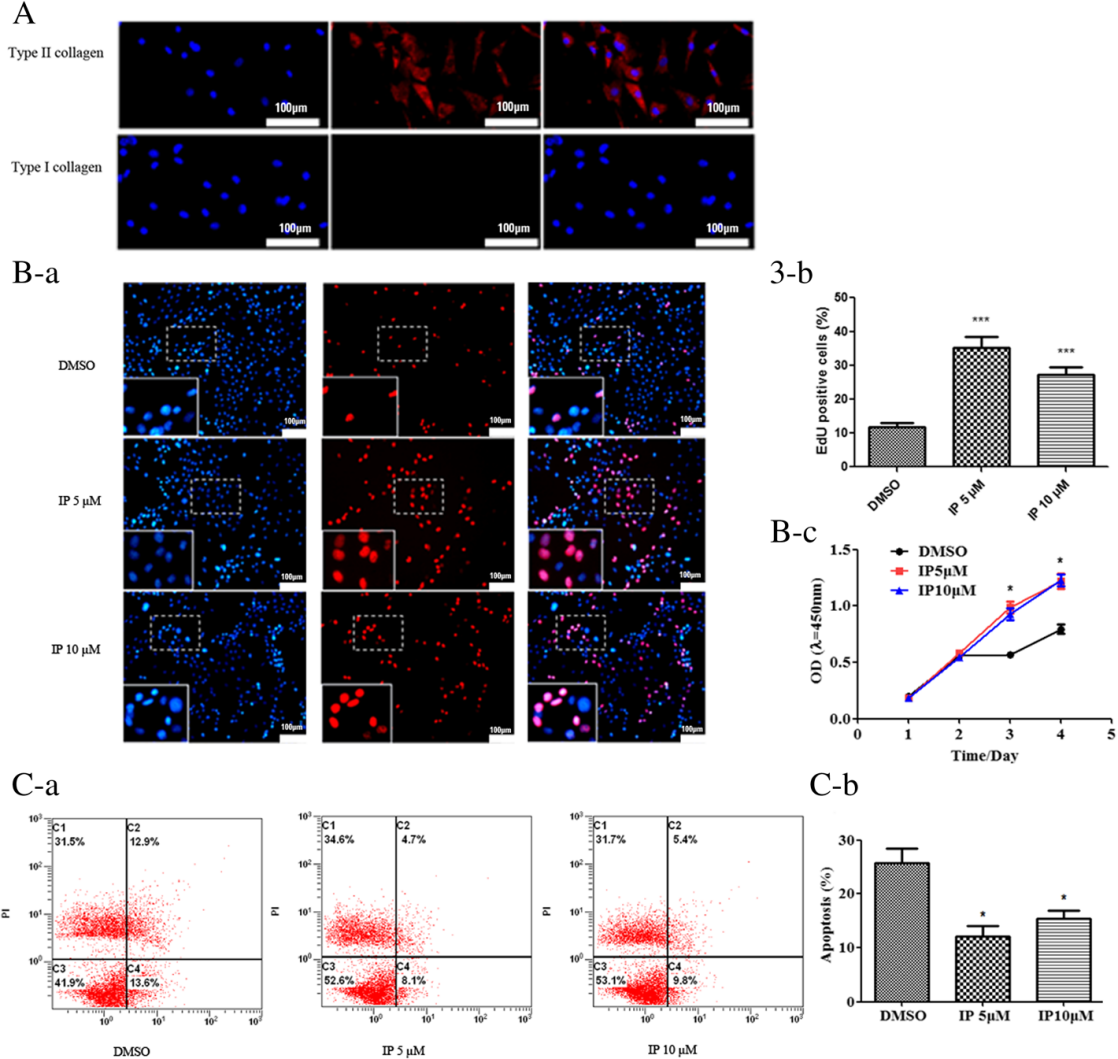
Ipriflavone promoted the proliferation and reduced the apoptosis of human chondrocytes. A, The results of the immunofluorescence assay showed that the cells (primary human chondrocytes were incubated in DMEM supplemented with 10% FBS in the presence or absence of ipriflavone) were positive for type II collagen and negative for type I collagen staining (red, scale bar: 100 μm), and the cultured cells maintained their chondrocytes phenotype. B-a, The EdU-based cell proliferation assay showed that compared with the DMSO group, and the EdU-positive cells (red, scale bar: 100 μm) were significantly increased in both the 5 μM and 10 μM IP treatment groups. B-b, The percentage of EdU-positive cells was quantified, and the proliferation of chondrocytes was significantly increased in both IP treatment groups. Data are expressed as means ± SDs (*n* = 6) ***P < 0.001 versus the DMSO group. B-c, The CCK-8 assay results showed that the viability of chondrocytes was higher in both IP treatment groups than the DMSO control group, and the viability gradually increased with a longer treatment time. Thus, IP significantly promotes the proliferation of human chondrocytes in vivo. C, The Annexin V-FITC/propidium iodide (PI) dual staining assay by flow cytometry indicated that apoptosis was reduced in the IP treatment group compared with the DMSO control group at 48 h after treatment. Values are the mean ± SDs. (*n* = 3) *P < 0.05 versus the DMSO group

This study was approved by the Institutional Animal Care and Use Committee of the Second Hospital of Shanxi Medical University (CMTT#: 2013025). Cartilage slices were removed from the “relatively normal” cartilage samples of the tibia obtained during total knee arthroplasty and washed in Dulbecco’s modified Eagle’s medium (DMEM) (Invitrogen, Carlsbad, CA, USA). Chondrocytes were isolated as previously described [24]. Briefly, pieces of cartilage were minced with a scalpel and digested with pronase (2 mg/ml) (Roche, Basel, Switzerland) in Hank’s Balanced Salt Solution (HBSS) (Invitrogen, Carlsbad, CA, USA) for 30 min at 37 °C with shaking. After digestion and removal of the supernatant, the cartilage pieces were washed with DMEM and digested with crude bacterial collagenase (type IA, 1 mg/ml) (Sigma-Aldrich, St Louis, MO, USA) for 6–8 h at 37 °C with shaking. Enzymatic digestion was stopped by adding DMEM containing 10% fetal bovine serum (FBS) (Invitrogen, Carlsbad, CA, USA). Residual multicellular aggregates were removed by filtering, and the cells were washed three times with DMEM. Primary chondrocytes were incubated in DMEM containing 10% FBS, l-glutamine (Invitrogen, Carlsbad, CA, USA), and antibiotics (penicillin and streptomycin) (Sigma-Aldrich, St Louis, MO, USA) under 37 °C, 5% CO_2_ condition and allowed to attach to the surface of the culture dishes (Nalge Nunc International Corp, Naperville, IL, USA). At 90% confluence, the cells were treated with ipriflavone (Sigma-Aldrich, St Louis, MO, USA) dissolved in dimethyl sulfoxide (DMSO) (Sigma-Aldrich, St Louis, MO, USA) to a concentration of 5 μM and 10 μM, and the 0.1% DMSO treatment group was used as the control. After 48 h in culture without removing the reagent, the cell proliferation assay, cell apoptosis assay, total RN, and total protein were isolated from the chondrocytes. Immunocytochemistry analyses of chondrocyte phenotypes were performed as described previously using anti-type I collagen (SC-59772, Santa Cruz, CA, USA) and anti-type II collagen monoclonal antibodies (SC-52658, Santa Cruz, CA, USA) [25]. These cells were positive for type II collagen and negative for type I collagen staining (Fig. 1A). In addition, cell proliferation and apoptosis assays were also conducted using chondrocytes.

### Human cartilage explant culture

Cartilage samples were obtained from the “relatively normal” cartilage samples of the tibial plateau obtained during total knee arthroplasty. The cartilage samples were then cut into 4 mm^3^ pieces weighing 6–9 mg each with a scalpel and cultured in DMEM/F12 supplemented with 10% FBS. Among the collected explants, we randomly subdivided them into three groups: treatment with 50 μM ipriflavone, 100 μM ipriflavone, and 0.1% DMSO as the control (Fig. 3A). After 72 h of treatment without removing the reagent, the total mRNA and total protein were isolated from the cartilage tissues, respectively.

### Rat anterior cruciate ligament transection (ACLT) model of OA treated with ipriflavone

Two-month-old male SD rats (180–230 g, *n* = 75) were obtained from the Experimental Animal Centre, Shanxi Medical University, China. The rats were housed under specific pathogen-free conditions with a 12-h/12-h light/dark cycle. All animals were handled and used in accordance with the Guidelines for the Use and Care of Laboratory Animals provided by Shanxi Medical University. All animal experiments were approved by the Institutional Review Board at the Second Hospital of Shanxi Medical University (Taiyuan, China; CMTT#: 2013012, Approval 2013).

The rats were randomized to 3 groups (*n* = 25 per group): (1) sham operation, (2) ACLT alone, and (3) ACLT and 200 mg/kg ipriflavone [21, 22]. ACLT and sham operations were performed on the right knees as described previously [26]. Ipriflavone was used to treat rat posttraumatic osteoarthritis (PTOA) by intragastrical administration. The treatment was performed 24 h after ACLT and then once a day for 6 weeks. All animals were euthanized 12 weeks after the operation. Downstream genes of Ihh signaling and OA-related gene expression were quantified by real-time polymerase chain reaction (PCR). The extent of OA progression was graded using Safranin-O and immunohistochemical staining. Ten rats were used to detect cartilage degeneration, and 15 rats were assessed using real-time PCR in each group.

### Histology

Seventy-two hours after culture with ipriflavone or 0.1% DMSO, the human cartilage explants were fixed in 10% formalin (Sigma Aldrich, St Louis, MO, USA) for 72 h. The specimens were decalcified in Richman-Gelfand-Hill solution, processed in a Tissue-Tek VIP 1000 tissue processor (Miles, Elkhart, IN, USA), and embedded in a single block of Paraplast X-tra (Thermo-Fisher, Hampton, NH, USA). Blocks were trimmed to expose the tissue using a rotary microtome (Leica RM2125, Leica Microsystems Ltd., Wetzlar, Germany). Ten adjacent sections were collected at intervals of 0 μm, 100 μm, and 200 μm. Two serial 6-μm-thick sections from each interval were stained with Safranin O.

In the in vivo study, the femurs and tibiae were hemisected in the midsagittal plane, and each half was embedded in a single block of Paraplast X-tra (Thermo-Fisher, Hampton, NH, USA). Blocks were trimmed to expose the cartilage. Ten adjacent sections were collected at intervals of 0 μm, 100 μm, and 200 μm (Leica RM2125, Leica Microsystems Ltd., Wetzlar, Germany). Two serial 6-μm-thick sections from each interval were stained with Safranin O. Cartilage degradation was quantified by two independent and blinded observers using an Osteoarthritis Research Society International (OARSI) grading system [27].

### Immunohistochemistry

To detect the distribution of type II collagen, MMP-13, type X collagen, and type II collagen breakdown product (type II collagen-C fragment) in cartilage, we carried out immunohistochemistry using the 3,3′-diaminobenzidine (DAB) Histostain streptavidin-peroxidase (SP) kit (Novex, Life Technologies, USA). The sections (6 μm) were deparaffinized and rehydrated using conventional methods. The sections were digested with 5 mg/ml of hyaluronidase in phosphate-buffered saline (PBS; Sigma) at 37 °C for 10 min. Endogenous peroxidase was blocked by treating the sections with 3% hydrogen peroxide in methanol (Sigma-Aldrich) at room temperature for 10 min. Nonspecific protein binding was blocked by incubation with a serum blocking solution (Li-Cor) at room temperature for 10 min. The sections were incubated with specific antibodies against rat type II collagen, MMP-13, types X collagen, or type II collagen-C fragment (IBEX Technologies, Mont-Royal, QC, Canada) at 4 °C overnight. Thereafter, the sections were treated sequentially with biotinylated secondary antibody and SP conjugates at 37 °C for 10 min and then developed in DAB chromogen (Invitrogen) for 3 min. The sections were counterstained with hematoxylin (Invitrogen) for 1 min. Photomicrographs were obtained with a Nikon E800 microscope (Nikon, Melville, NY, USA).

### Western blotting

The total protein of chondrocytes and cartilage explants was isolated and quantified using the BAC Protein Assay Reagent Kit (Pierce, Rockford, IL, USA). Fifteen micrograms of total protein was electrophoresed by 10% SDS PAGE under reducing conditions. After electrophoresis, the proteins were transferred onto an NC membrane (Beyotime Biotechnology, China) and probed with a polyclonal antibody against Smo (SC-166685, Santa Cruz, CA, USA), type II collagen, MMP-13 (SC-52658, SC-515284 Santa Cruz, CA, USA), type X collagen (ab49945 Abcam, USA), and Runx-2 (SC-390351 Santa Cruz, CA, USA). The antibody was diluted 1:500 in PBS-T containing 1% bovine serum albumin (BSA) (Sigma-Aldrich, St Louis, MO, USA). Horseradish peroxidase-conjugated secondary antibody IgG (H + L) (SC-2371, Santa Cruz, CA, USA) was diluted 1:2000 in PBS-T. Visualization of immunoreactive proteins was achieved using ECL Western blotting detection reagents (KWBIO, Beijing, China) and subsequently exposing the membrane to Kodak X-Omat AR film (Kodak, Rochester, NY). Band densities were quantified using Image Acquisition and Analysis Software (UVP, Upland, CA, USA).

### Real-time quantitative PCR

The total RNA was isolated from human chondrocytes and rat knee joint cartilage using an RNeasy isolation kit (Takara, Japan). Cartilage samples (tibial plateau and femur condyle) from three rats were dissected using a scalpel and pooled together; there were 5 pooled samples per group. Total RNA (1 μg) was reverse-transcribed to complementary DNA (cDNA) using a reverse transcription kit (Takara, Japan). The resulting cDNA (80 ng/μl) was used as the template to quantify the relative content of messenger RNA (mRNA) using a QuantiTect SYBR Green PCR kit (Takara, Japan) with an IQ5 Fluorescence Detection System (Bio-Rad). Primer pairs were listed in Table 1. Relative transcript levels were calculated according to the equation *x* = 2^−⊿⊿Ct^, where ΔΔC_t_ = ΔC_t_ E-ΔC_t_ C(ΔC_t_ E = C_t_ exp- C_t_ 18S and ΔC_t_ C = C_t_ C- C_t_18S) [6].

**Table 1:** Primers used in this paper with their species, name, orientation, and sequence used in the RT-PCR protocol

Species	Name	Forward/reverse	Sequence
Human	Col2a1	Forward	TGA GGG CGC GGT AGA GAC CC
	Col2a1	Reverse	TGC ACA CAG CTG CCA GCC TC
	Col10a1	Forward	TGC CTC TTG TCA GTG CTA ACC
	Col10a1	Reverse	GCG TGC CGT TCT TAT ACA GG
	MMP-13	Forward	TGC TGC ATT CTC CTT CAG GA
	MMP-13	Reverse	ATG CAT CCA GGG GTC CTG GC
	Runx-2	Forward	GGC AGG CAC AGT CTT CCC
	Runx-2	Reverse	GGC CCA GTT CTG AAG CAC C
	Smo	Forward	CCT TTG GCT TTG TGC TCA TTA CCT T
	Smo	Reverse	CGT CAC TCT GCC CAG TCA ACC T
	Gli1	Forward	GAA CCC TTG GAA GGT GAT ATG TC
	Gli1	Reverse	GGC AGT CAG TTT CAT ACA CAG AT
	Gli2	Forward	GCG TGT TTA CCC AAT CCT GT
	Gli2	Reverse	GAT GCT CCC TCA GAG TCC TG
	Hhip	Forward	TCC GGT CAC ATC TTG GGA TT
	Hhip	Reverse	GTC TGT GCA GGT TGT ACC GTG
	Ptc1	Forward	ATG CTG GCG GGA TCT GAG TTC GAC T
	Ptc1	Reverse	GGG TGT GGG CAG GCG GTT CAA G
	Gli3	Forward	CTT TGC AAG CCA GGA GAA AC
	Gli3	Reverse	TTG TTG GAC TGT GTG CCA TT
Rat	Col2	Forward	GAG GGC AAC AGC AGG TTC AC
	Col2	Reverse	TGT GAT CGG TAC TCG ATG ATG G
	Agg	Forward	CAG TGC GAT GCA GGC TGG CT
	Agg	Reverse	CCT CCG GCA CTC GTT GGC TG
	Col10	Forward	CCA GGT GTC CCA GGA TTC CC
	Col10	Reverse	CAA GCG GCA TCC CAG AAA GC
	MMP13	Forward	GGA CCT TCT GGT CTT CTG GC
	MMP13	Reverse	GGA TGC TTA GGG TTG GGG TC
	Smo	Forward	TCT CGG GCA AGA CAT CCT
	Smo	Reverse	TAG CCT CCC ACA ATA AGC A
	Gli-1	Forward	GCC AAT CAC AAA TCA GTC TCC
	Gli-1	Reverse	TGC TCC TAA CCT GCC CAC
	Gli-2	Forward	AGG CCC AGT ACA TGC TGG TTG
	Gli-2	Reverse	GGA CCG CAG GTG TGT CTT CA
	Gli-3	Forward	TGG GAT TCC GAC GGT TCT G
	Gli-3	Reverse	GGA GGT CTT CAT CGG GCT TG
Human and rat	18S	Forward	CGG CTA CCA CAT CCA AGG AA
	18S	Reverse	GCT GGA ATT ACC GCG GCT

### Cell proliferation assays

Cell proliferation was detected using the Cell Counting Kit-8 (CCK-8) cell viability assay kit (Boster Biological Technology, China) and Cell-Light™ EdU Kit (RiboBio, Guangzhou, China) according to the manufacturer’s protocol. The CCK-8 assay was performed to detect the proliferation of chondrocytes treated with ipriflavone for 1 day, 2 days, 3 days, and 4 days. The cell suspension (100 μl/well) was added to a 96-well plate, and the plate was preincubated in a humidified incubator (at 37 °C, 5% CO_2_). Next, 10 μl of the CCK-8 solution was added to each well of the plate and incubated for 4 h in the incubator, followed by measuring the absorbance at 450 nm using microplate reader (Bio-Rad, CA, USA). EdU was added to the culture medium at a concentration of 50 μM. The cells were fixed with 4% paraformaldehyde, permeabilized with 0.5% Triton X-100 in PBS for 15 min, and subsequently incubated with Apollo® reaction cocktail (containing Apollo® reaction buffer, Apollo® catalyst, Apollo® 567 fluorescent dyes and buffer additives) and Hoechst 33342, for 30 min away from light. The cells were then observed immediately under a fluorescence microscope (Olympus, Japan). The percentage of positive cells (red) was determined.

### Cell apoptosis assay by flow cytometry

Cell apoptosis was detected using a Cell Apoptosis Assay Kit (BOSTER Biological Technology, China) according to the manufacturer’s protocol. After 48 h in culture with ipriflavone or DMSO, chondrocytes were digested with 0.25% trypsin and dissociated into single cells, followed by double-staining with fluorescein FITC-labeled annexin V and propidium iodide (PI) in binding buffer (BD, USA) for 15 min away from light. Finally, apoptosis was detected by flow cytometry (FCM, BD, USA). The experiment was repeated three times. Cells that were positive for annexin V alone were recognized as early apoptosis, cells that were positive for PI only were recognized as necrosis, and cells that were positive for both annexin V and PI were recognized as late apoptosis.

### Statistical analysis

The data represent the means ± SD obtained from at least three independent experiments. Each experimental measure was performed in triplicate. Two-tailed paired *t* tests were used to compare the changes in gene expression levels and protein expression levels of Smo, Gli-1, − 2, − 3, Runx-2, type II collagen, type X collagen, and MMP-13. *P* values less than 0.05 were considered significant. Statistical analyses were performed using SPSS software.

## Results

### Ipriflavone promoted the proliferation and reduced the apoptosis of human chondrocytes

To determine the effect of ipriflavone on the proliferation of chondrocytes, an EdU-based cell proliferation assay and CCK-8 assay were performed. As revealed by EdU cell proliferation staining (red) (Fig. 1B-a), ipriflavone significantly promoted chondrocyte proliferation. Approximately 300 cells from 3 independent experiments were scored, and the percentages of EdU-positive cells in the 5 μM and 10 μM ipriflavone groups were 36.40% ± 1.32% and 28.54% ± 0.74%, respectively (*P* < 0.001), whereas human OA chondrocytes treated with DMSO had the lowest EdU-positive stained cells (11.99% ± 0.35%) (Fig. 1B-b). The CCK-8 assay results showed that the viability of chondrocytes was higher in the ipriflavone treatment groups than the DMSO control group, and the viability gradually increased with a longer treatment time (Fig. 1B-c). To further determine the effect of ipriflavone on chondrocyte apoptosis, we performed an annexin V-FITC/propidium iodide (PI) dual staining assay by flow cytometry, and the results showed that apoptosis was reduced in the ipriflavone treatment group than the DMSO control group after 48 h of treatment (Fig. 1C-a). To confirm the above results, the cellular apoptosis rate was measured. The results demonstrated that the percentage of apoptotic cells in the DMSO, 5 μM or 10 μM ipriflavone groups was 25.76% ± 5.1%, 12.64% ± 3.7%, and 15.18% ± 3.13%, respectively (*P* < 0.05) (Fig. 1C-b). These findings suggested that ipriflavone was able to increase the proliferation and decrease the apoptosis of chondrocytes in vitro.

### Ipriflavone downregulated OA-related gene and protein expression in human chondrocyte culture by inhibiting Ihh signaling

**Fig. 2:**
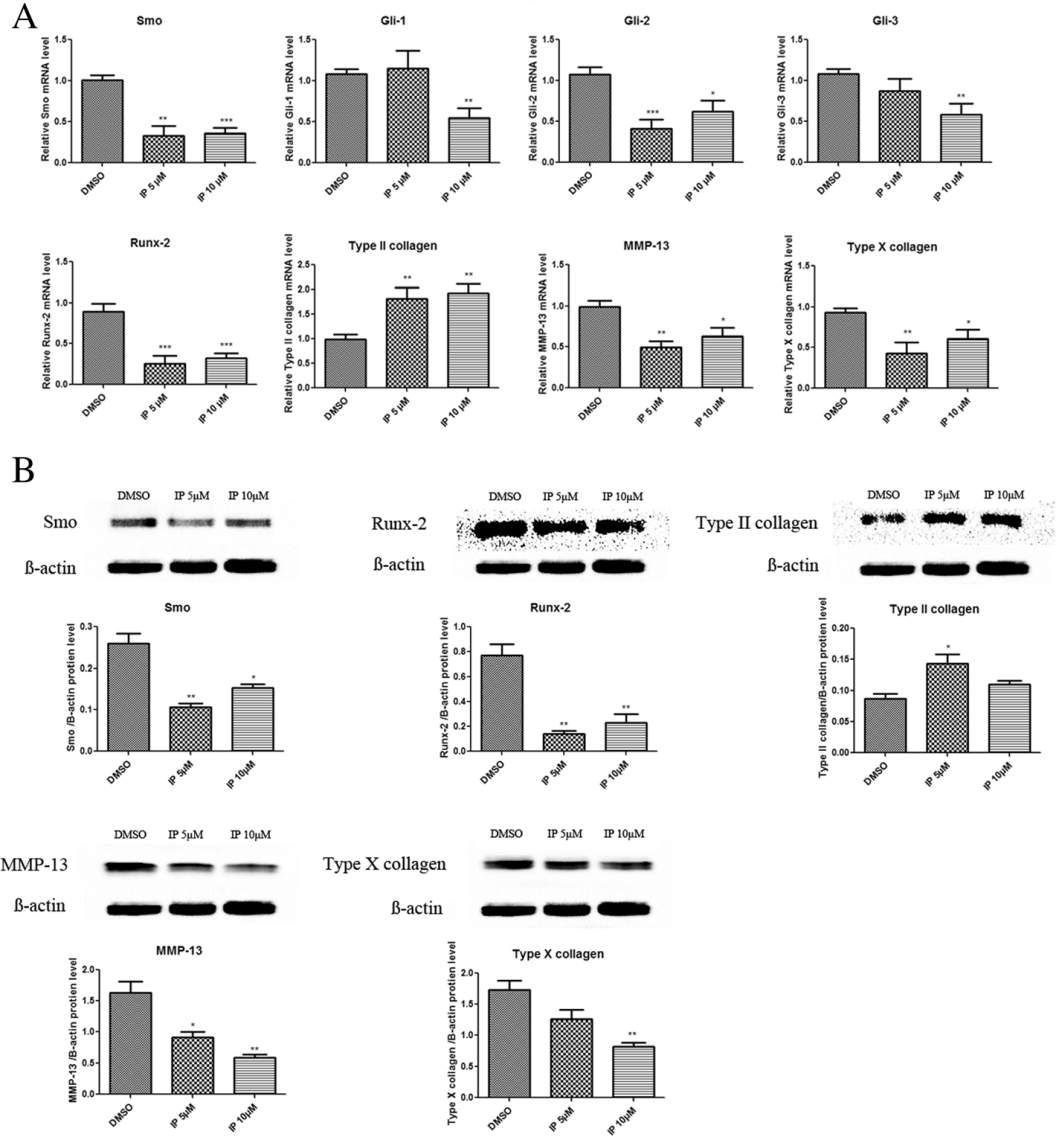
Chondroprotective effect of ipriflavone (IP) in human chondrocytes. **a** Real-time PCR results showed reduced mRNA expression of key genes in the Ihh pathway, Smo, Gli-1,Gli-2,Gli-3, and Runx-2, at 48 h after IP treatment, and among the three kinds of Glis, the reduction of Gli-2 was especially significant. The MMP-13 and type X collagen mRNA levels were decreased, and the type II collagen mRNA level was significantly increased in human chondrocytes. **b** Western blot results indicated that in chondrocytes, the expression of Smo and Runx-2 protein was decreased at 48 h after IP treatment, MMP-13 and type X collagen expression was decreased in the IP treatment group, and type II collagen expression was increased in the IP treatment group. The gray value of the Western blot bands was semiquantified using Image Analysis Software (Image Lab 3.0). Values are the mean ± SEM. *n* = 3, *P < 0.05, **P < 0.01, ***P < 0.001 versus the DMSO group

The results of real-time PCR indicated that ipriflavone significantly decreased the mRNA levels of key genes in the Ihh signal pathway (Smo, Gli2, Runx-2) at both 5 μM and 10 μM after 48 h of treatment; however, the mRNA levels of Gli1 and Gli3 were decreased only in the 10 μM ipriflavone treatment group. Ipriflavone also decreased the expression of MMP-13 and type X collagen mRNA and increased the expression of type II collagen mRNA in both ipriflavone groups (Fig. 2A). The Western blotting results showed that compared with the DMSO control group, the expression of key proteins in Ihh signaling (Smo and Runx-2) were significantly decreased in both the 5 μM and 10 μM ipriflavone treatment groups after 48 h, and the expression of MMP-13 and type X collagen was also significantly decreased at both concentrations. Simultaneously, the expression of type II collagen was significantly increased (Fig. 2B). These results suggested that ipriflavone had a chondroprotective effect by decreasing OA-related gene and protein expression and increasing the expression of anabolic factors by inhibiting the Ihh pathway.

### Ipriflavone reduced the degeneration of cartilage by inhibiting Ihh signaling in cultured human cartilage explants

**Fig. 3:**
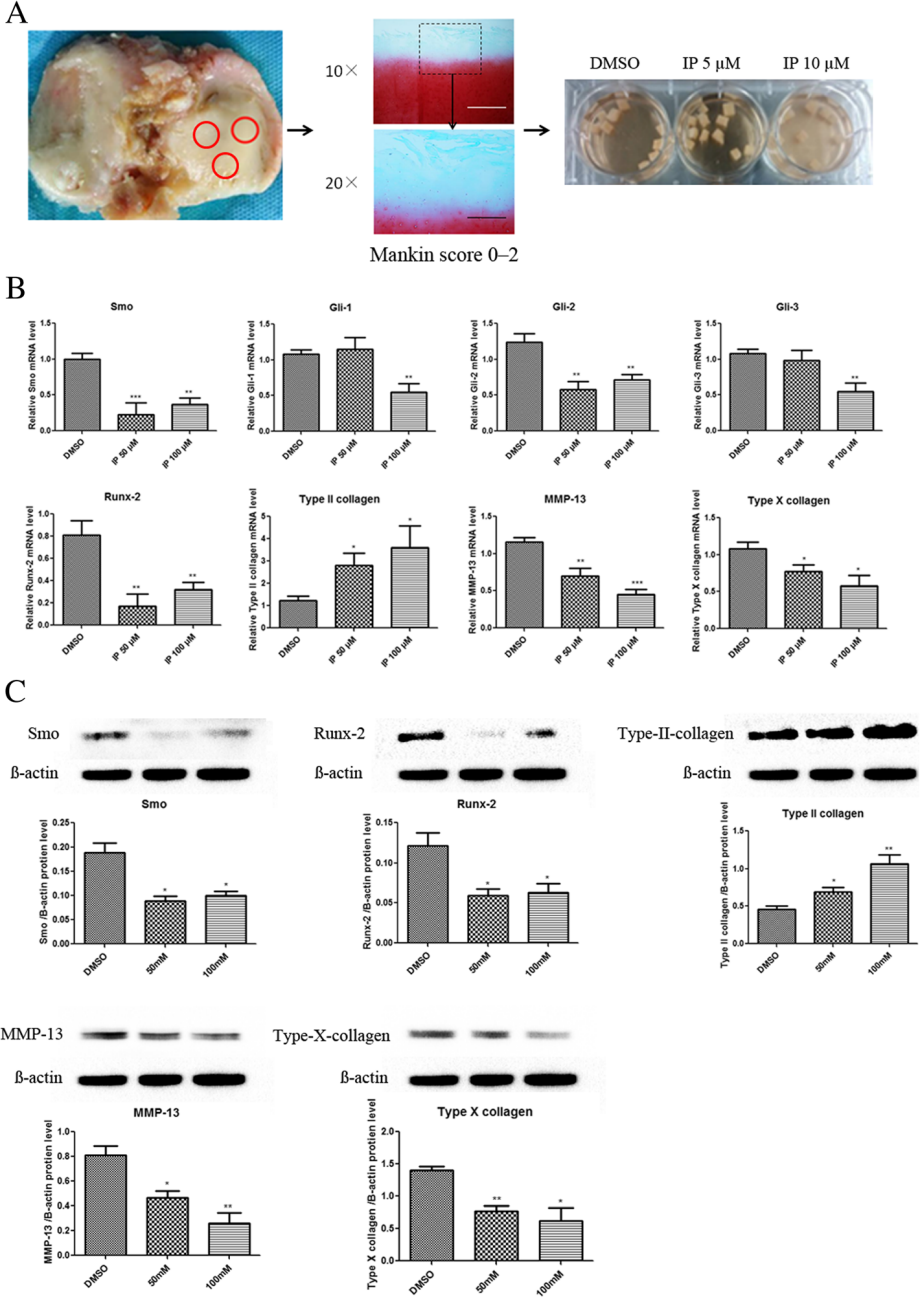
Ipriflavone reduced the degeneration of cartilage by inhibiting Ihh signaling in cultured human cartilage explants. **a** Safranin O staining results showed that cartilage samples were taken from “relatively normal” cartilage samples of the tibia obtained during total knee arthroplasty (Mankin score 0–2). Then, the cartilage samples were cut into 4-mm^3^ pieces weighing 6–9 mg each and randomly subdivided into three groups: 0.1% DMSO group, 50 μM IP treatment group, and 100 μM IP treatment group. **b** Real-time PCR results showed that the mRNA expression of key genes in Ihh pathway, Smo, Gli-1,Gli-2,Gli-3, and Runx-2 was decreased in the IP treatment group, especially the reduction of Gli-2. Simultaneously, the mRNA levels of type II collagen was increased while MMP-13 and type X collagen were decreased in both IP treatment groups. **c** Western blotting results indicated that the expression of key proteins in Ihh signaling (Smo and Runx-2) were decreased in the IP treatment groups compared with the DMSO control group. MMP-13 and type X collagen protein were also reduced, while the expression of type II collagen protein was increased compared with the DMSO control group. Values are the mean ± SEM. *n* = 3, *P < 0.05, **P < 0.01 versus the DMSO and IP treatment group

To confirm the results from the monocultures, human cartilage explants (4 mm^3^ pieces) were treated with 50 μM ipriflavone, 100 μM ipriflavone, and DMSO. After 72 h in culture without removing the reagent, the total mRNA and total protein were isolated from the cartilage tissue to detect the expression of key genes and proteins, respectively. Real-time PCR results showed that the mRNA levels of Smo, Gli-2, and Runx-2 were decreased in both ipriflavone treatment groups. Type II collagen mRNA levels were increased while MMP-13 and type X collagen mRNA levels were decreased in both ipriflavone treatment groups (Fig. 3B). Western blotting results indicated that the expression of key proteins in Ihh signaling (Smo and Runx-2 protein) were decreased in both ipriflavone treatment groups compared with the DMSO control group. MMP-13 and type X collagen proteins were also reduced, while the expression of type II collagen protein was higher than in the DMSO control group (Fig. 3C).

### Ipriflavone attenuated the degeneration of articular cartilage by blocking the Indian hedgehog pathway in rat OA models

**Fig. 4:**
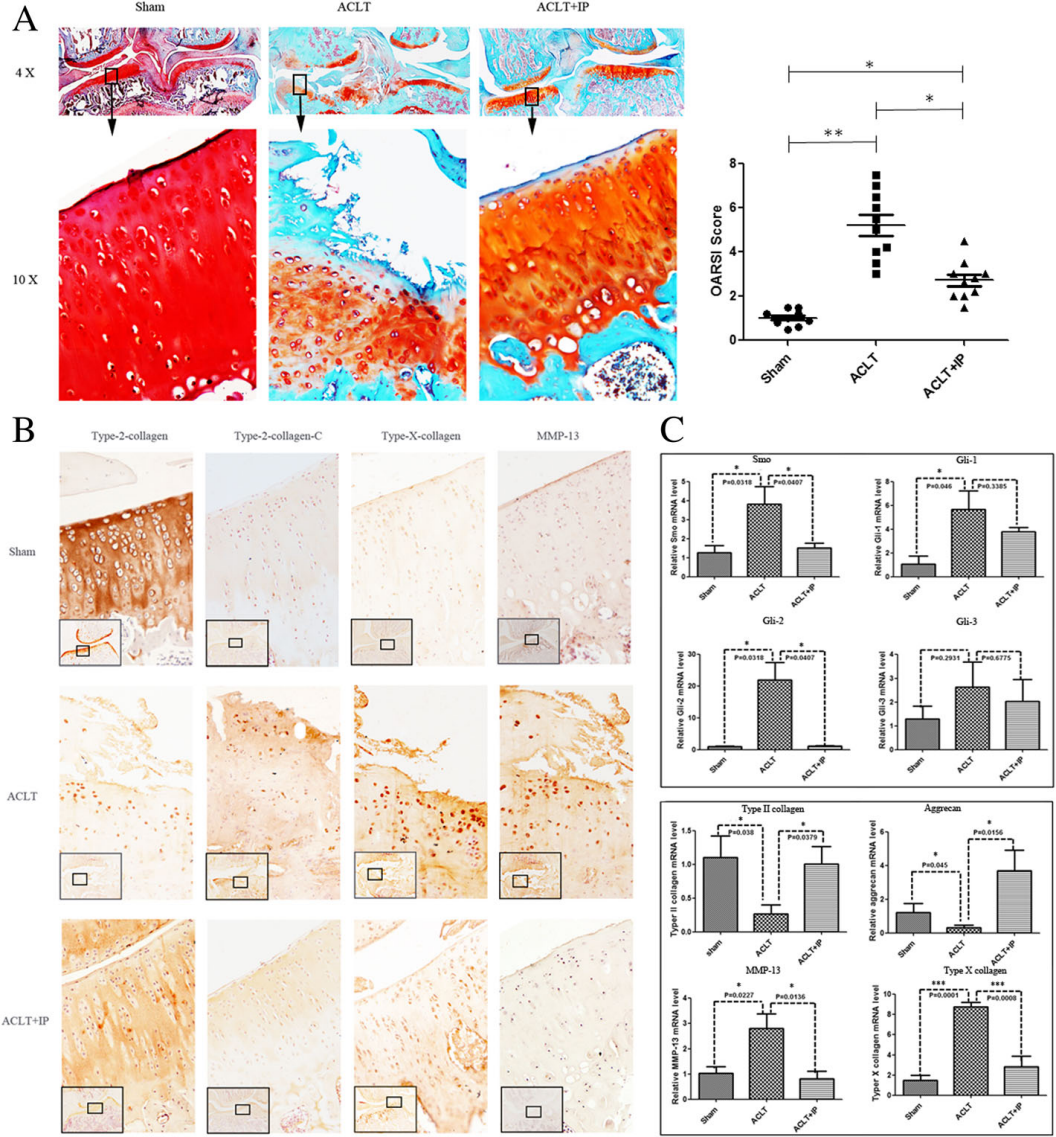
Ipriflavone (IP) reduced the degeneration of cartilage by blocking the Indian hedgehog pathway in vivo. **a** The results of Safranin O staining indicated that there was less surface damage with stronger Safranin O staining in articular cartilage specimens from IP-treated animals compared with the ACLT group at 12 weeks after the operation. Cartilage destruction was quantified and compared with the ACLT group, and the summed OARSI scores were significantly decreased in the IP treatment group. Data are expressed as means ± SDs. (*n* = 10), *P < 0.05 **P < 0.01. **b** Type II collagen expression in articular cartilage was higher in IP-treated and sham-operated rats than in rats that underwent ACLT without treatment. In contrast, degraded collagen II (type II collagen-C fragment), type X collagen, and matrix metalloproteinase 13 (MMP-13) staining levels were elevated in rats that underwent ACLT without treatment with respect to the IP-treated and sham-operated rats, which is consistent with reduced OA damage. **c** Real-time PCR results indicated that the expression of key genes in the Ihh pathway (Smo, Gli-1, Gli-2, Gli-3) was increased in rats that underwent ACLT without IP treatment and reduced in IP-treated rats at 12 weeks after the ACLT operation, especially Smo and Gli-2. Concomitantly, type II collagen and Agg expression in articular cartilage was elevated in IP-treated and sham-operated rats compared with rats that underwent ACLT without treatment. In contrast, type X collagen and matrix metalloproteinase 13 (MMP-13) were elevated in rats that underwent ACLT without IP treatment, and their expressions were reduced in IP-treated rats at 12 weeks after the ACLT operation, consistent with the reduced OA damage. Values are the mean ± SEM. *P* < 0.05 versus the ACLT and IP treatment group

To further test ipriflavone in vivo, it was intragastrically administered in the rat ACLT model of OA (200 mg/kg). Cartilage degeneration was analyzed after 12 weeks of treatment by histology. Safranin O staining demonstrated that there was less surface damage with stronger Safranin O staining in articular cartilage specimens from ipriflavone-treated animals compared with ACLT group animals. Osteoarthritis scores (OARSI scores) were significantly reduced in the ipriflavone treatment group compared with the ACLT group (Fig. 4A). The results of immunohistochemical staining indicated that type II collagen expression in articular cartilage was higher in the ipriflavone-treated and sham-operated rats than in rats that underwent ACLT without treatment. In contrast, degraded collagen II (type II collagen-C fragment), type X collagen, and matrix metalloproteinase 13 (MMP-13) staining were elevated in rats that underwent ACLT without treatment with respect to the ipriflavone-treated and sham-operated rats (Fig. 4B), which is consistent with the reduced OA damage. Real-time PCR results indicated that the expressions of key genes in the Ihh pathway (Smo, Gli-1, Gli-2, Gli-3) were increased in rats that underwent ACLT without IP treatment, and they were reduced in the ipriflavone-treated rats at12 weeks after the ACLT operation, especially Smo and Gli-2. Simultaneously, type II collagen and Agg expression in articular cartilage was higher in the ipriflavone-treated and sham-operated rats than in rats that underwent ACLT without treatment. In contrast, type X collagen and MMP-13 were elevated in rats that underwent ACLT without ipriflavone treatment, and the expression levels were reduced in ipriflavone-treated rats at 12 weeks after the ACLT operation (Fig. 4C), which is consistent with the reduction of OA damage by blocking Ihh signaling.

## Discussion

Ihh is a secreted protein that is expressed in prehypertrophic chondrocytes [28]. This protein is highly important in the regulation of hypertrophic differentiation of articular chondrocytes during endochondral ossification [29, 30]. Studies have shown that Ihh can bind Patched-1 (PTCH1) receptor, relieving Smoothened (Smo) inhibition and activating the glioma-associated oncogene homolog (GLI) family (Gli1/2/3), which will upregulate the gene expression of Runt-related transcription factor 2 (Runx-2) and promote chondrocyte hypertrophy [3–5].

Articular chondrocytes become hypertrophic in OA cartilage [31, 32]. The collective evidence indicates that Ihh plays a critical role in OA cartilage degeneration and that Ihh blockade may have a chondroprotective effect for OA treatment. However, Ihh inhibitors such as cyclopamine, the first Smo antagonist, cause serious side effects, including weight loss and dehydration [33, 34], holoprosencephaly (HPE), cleft lip palate (CLP), and limb defects in vivo [9–13]. To overcome these side effects, some modified cyclopamine prodrugs have been developed, such as IPI-269609 and IPI-926 [35, 36], but these drugs are only used as anticancer agents and still exhibit high toxicity to other tissues and organs, limiting the broad use of inhibitors of the Ihh pathway in humans. The results of our study demonstrate, for the first time, that ipriflavone, a novel and safe inhibitor of Ihh signaling, can attenuate cartilage degeneration by blocking the Ihh pathway in vitro and in vivo.

Our in vitro and in vivo data clearly demonstrated the following: (1) Ipriflavone promoted the proliferation and reduced the apoptosis of human chondrocytes. (2) Ipriflavone inhibited the expression of target gene Runx-2 by Smo-Gli2 pathway mainly. (3) The expression of OA-related genes and proteins, including MMP-13 and type X collagen, were reduced while type II collagen was increased after ipriflavone treatment in vitro and in vivo, indicating that ipriflavone is a promising therapeutic drug to inhibit cartilage degeneration by blocking Ihh signaling. Our results are in agreement with our previous study [16] and the findings of others [10], which reported that inhibition of Ihh signaling has the potential to prevent surgically induced OA in transgenic mice. These findings suggest that ipriflavone may be a safe and effective oral medicine for the treatment of OA.

Dose-response assays of ipriflavone in a previous study suggested that concentrations of ipriflavone between 1 μM and 10 μM can inhibit the Ihh signaling pathway, and the inhibition rates of 1 μM and 10 μM ipriflavone were approximately 20% and 100%, respectively [14]. In our pilot study, we treated chondrocytes with 1 μM, 2.5 μM, 5 μM, and 10 μM ipriflavone. The results showed that the gene expression of Smo, the key downstream gene of the Ihh pathway, was blocked significantly in the 5 μM and 10 μM groups (Additional file 1: Figure S1). We further measured the proliferation and apoptosis of the chondrocytes in the 5 μM and 10 μM groups, and the results indicated significantly increased proliferation and decreased apoptosis in the two ipriflavone treatment groups, which demonstrated that these two doses are safe in human chondrocytes. Interestingly, upon treating human chondrocytes with ipriflavone and Ihh recombinant protein (5.0 μg/ml [6]), we observed a change in proliferation and apoptosis (Additional file 2: Figure S2), which indicated that the changes in cell proliferation and apoptosis induced by ipriflavone may be regulated by the inhibition of the Ihh signaling pathway. Promoting the proliferation of and inhibiting apoptosis of chondrocytes may be one of the mechanisms of the treatment of OA with ipriflavone.

A previous study reported that ipriflavone acts downstream of Ptc1 and upstream of Gli and that it acts similarly to cyclopamine [14]. However, the function of Gli1/2/3 in this process is still incompletely understood. Some studies have shown that Gli2 and Gli3 are essential for skeletal development, whereas Gli1 is not critical to this process; Gli1 acts synergistically with Gli2 and Gli3 [37]. Other studies have demonstrated that Gli1 and Gli2 seem to act mainly as activators of hedgehog target genes in the Ihh signaling pathway, and Gli3 may function both as a transcription factor and as a repressor of Ihh target genes [38, 39]. Our in vitro results indicated that only Gli2 was decreased in both IP groups, and our in vivo experiments showed similar results, specifically that only Gli2 was inhibited significantly; these findings suggest that ipriflavone inhibits Runx-2 mainly through the Smo-Gli2 pathway. In addition, we detected the mRNA expression of other relative genes of Ihh signaling, the results further confirmed our results too (Additional file 3: Figure S3). Our results are consistent with other reports [40].

The limitation of this study is that surgical transection of the ACL may not be as traumatic as an ACL injury sustained during physical activity. Bone bruises and chondral lesions frequently occur in the latter, and these concomitant injuries may also play a role in the development of posttraumatic OA. Nonetheless, the animal model of ACLT has been frequently used to study OA, and it mimics human OA both macroscopically and biochemically [41]. Minimizing local joint inflammation until ACL reconstruction is performed may be an important preventive measure that could forestall the long-term development of posttraumatic OA (Additional file 3).

## Conclusions

This study provides direct evidence that ipriflavone is able to attenuate cartilage degeneration by inhibiting Ihh signaling. Thus, ipriflavone could be a potential chondroprotective drug for OA treatment.

## Additional files

Additional file 1: Real-time PCR results showed reduced mRNA expression of key genes in the Ihh pathway, Smo, at 48 h after 5 μM and 10 μM groups but not in the cells treated with the 1 μM and 2.5 μM concentration. Values are the mean ± SEM. *n* = 3, **P < 0.01, versus the DMSO group. (TIF 117 kb)

Additional file 2: Human chondrocyte treated with ipriflavone and Ihh recombinant protein did change the level of the cell proliferation and apoptosis significantly. Primary chondrocytes were incubated in DMEM containing 10% FBS, under 37 °C, 5% CO_2_ condition. The cells were treated with 5 μM ipriflavone, 5 μM ipriflavone +Ihh (5.0 μg/ml) recombinant protein, respectively, and the 0.1% DMSO treatment group was used as the control. After 48 h in culture without removing the reagent, the cell proliferation assay and cell apoptosis assay were performed. A-a The EdU-based cell proliferation assay showed that compared with the DMSO group, the EdU-positive cells (red) were significantly increased in the 5 μM IP treatment group, and compared with the 5 μM IP treatment group, it was decreased in the 5 μM IP + Ihh treatment group significantly. A-b The percentage of EdU-positive cells was quantified, Data are expressed as means ± SDs (*n* = 3) ***P<0.001 versus the DMSO group, **P<0.01 versus the 5 μM IP treatment group. A-c The CCK-8 assay results showed that the viability of chondrocytes was higher in 5 μM IP treatment group than the DMSO control group, and the viability of chondrocytes was decreased by Ihh treatment. Data are expressed as means ± SDs (*n* = 3) **P<0.01, *P<0.05 versus the DMSO group. B-a The Annexin V-FITC/propidium iodide (PI) dual staining assay by flow cytometry indicated that apoptosis was reduced in the 5 μM IP treatment group compared with the DMSO control group at 48 h after treatment, and the reduction was blocked by Ihh treatment. Values are the mean ± SDs. (*n* = 3) *P < 0.05 versus 5 μM IP treatment group. (TIF 3873 kb)

Additional file 3: Real-time PCR results showed reduced mRNA expression of key genes in the Ihh pathway, Ptch1, and Hhip, at 48 h after 5 μM and 10 μM groups. Values are the mean ± SEM. *n* = 3, *P < 0.05 versus the DMSO group. (TIF 161 kb)

## Abbreviations

ACLT: Anterior cruciate ligament transection
Agg: Aggrecanase
BSA: Bovine serum albumin
CCK-8: Cell Counting Kit-8
DAB: 3,3′-Diaminobenzidine
DMEM: Dulbecco’s modified Eagle’s medium
DMSO: Dimethyl sulfoxide
FBS: Fetal bovine serum
Gli: Glioma-associated oncogene homolog
HBSS: Hank’s Balanced Salt Solution
Hh: Hedgehog
Ihh: Indian hedgehog
IP: Ipriflavone
MMP-13: Matrix metallopeptidase 13
OA: Osteoarthritis
OARSI: Osteoarthritis Research Society International
PBS: Phosphate-buffered saline
PTCH1: Patched-1
PTOA: Posttraumatic osteoarthritis
Runx-2: Runt-related transcription factor 2
Smo: Smoothened
SP: Streptavidin peroxidase
