# Supplementary figures and images for: Ipriflavone attenuates the degeneration of cartilage by blocking the Indian hedgehog pathway
Source: Arthritis Res Ther. 2019 May 2;21:109. doi: 10.1186/s13075-019-1895-x (PMC6498579; doi:10.1186/s13075-019-1895-x)

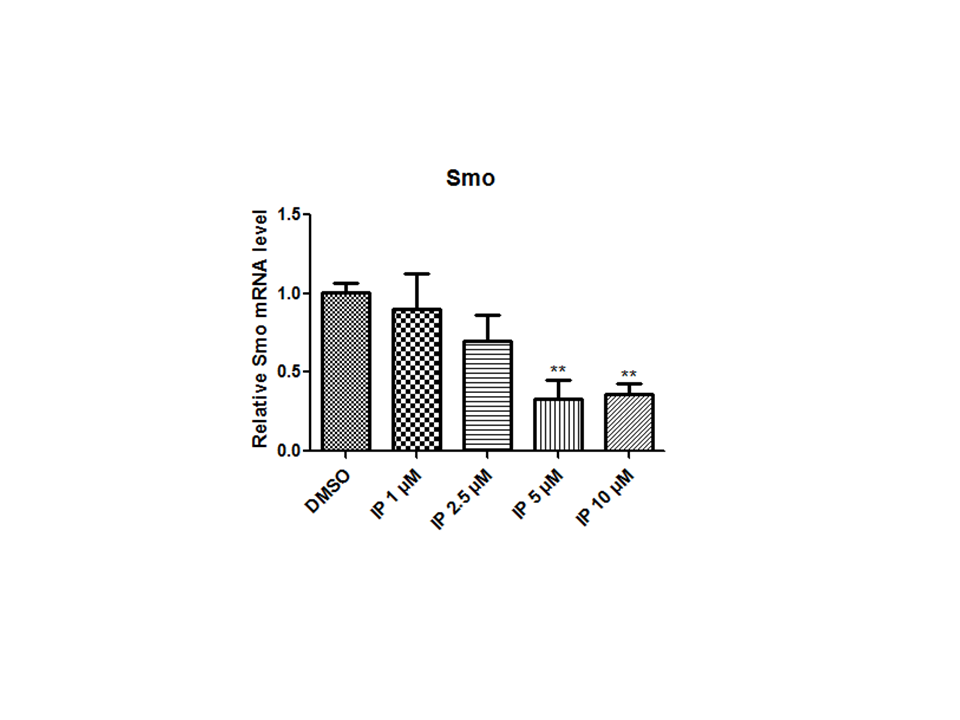

Supplement: Supplementary file 1 — Real-time PCR results showed reduced mRNA expression of key genes in the Ihh pathway, Smo, at 48 h after 5 μM and 10 μM groups but not in the cells treated with the 1 μM and 2.5 μM concentration. Values are the mean ± SEM. n = 3, **P < 0.01, versus the DMSO group. (TIF 117 kb) [file 13075_2019_1895_MOESM1_ESM.tif]

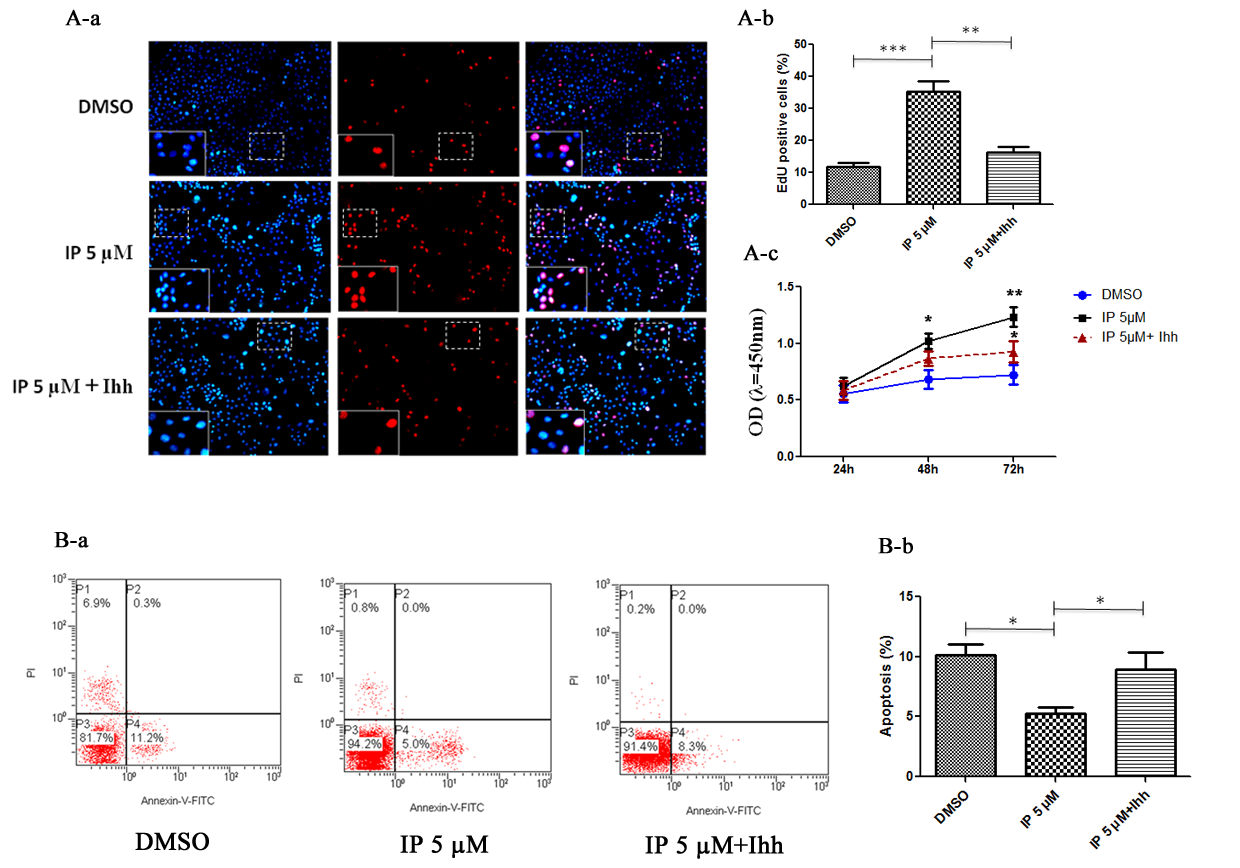

Supplement: Supplementary file 2 — Human chondrocyte treated with ipriflavone and Ihh recombinant protein did change the level of the cell proliferation and apoptosis significantly. Primary chondrocytes were incubated in DMEM containing 10% FBS, under 37 °C, 5% CO2 condition. The cells were treated with 5 μM ipriflavone, 5 μM ipriflavone +Ihh (5.0 μg/ml) recombinant protein, respectively, and the 0.1% DMSO treatment group was used as the control. After 48 h in culture without removing the reagent, the cell proliferation assay and cell apoptosis assay were performed. A-a The EdU-based cell proliferation assay showed that compared with the DMSO group, the EdU-positive cells (red) were significantly increased in the 5 μM IP treatment group, and compared with the 5 μM IP treatment group, it was decreased in the 5 μM IP + Ihh treatment group significantly. A-b The percentage of EdU-positive cells was quantified, Data are expressed as means ± SDs (n = 3) ***P<0.001 versus the DMSO group, **P<0.01 versus the 5 μM IP treatment group. A-c The CCK-8 assay results showed that the viability of chondrocytes was higher in 5 μM IP treatment group than the DMSO control group, and the viability of chondrocytes was decreased by Ihh treatment. Data are expressed as means ± SDs (n = 3) **P<0.01, *P<0.05 versus the DMSO group. B-a The Annexin V-FITC/propidium iodide (PI) dual staining assay by flow cytometry indicated that apoptosis was reduced in the 5 μM IP treatment group compared with the DMSO control group at 48 h after treatment, and the reduction was blocked by Ihh treatment. Values are the mean ± SDs. (n = 3) *P < 0.05 versus 5 μM IP treatment group. (TIF 3873 kb) [file 13075_2019_1895_MOESM2_ESM.tif]

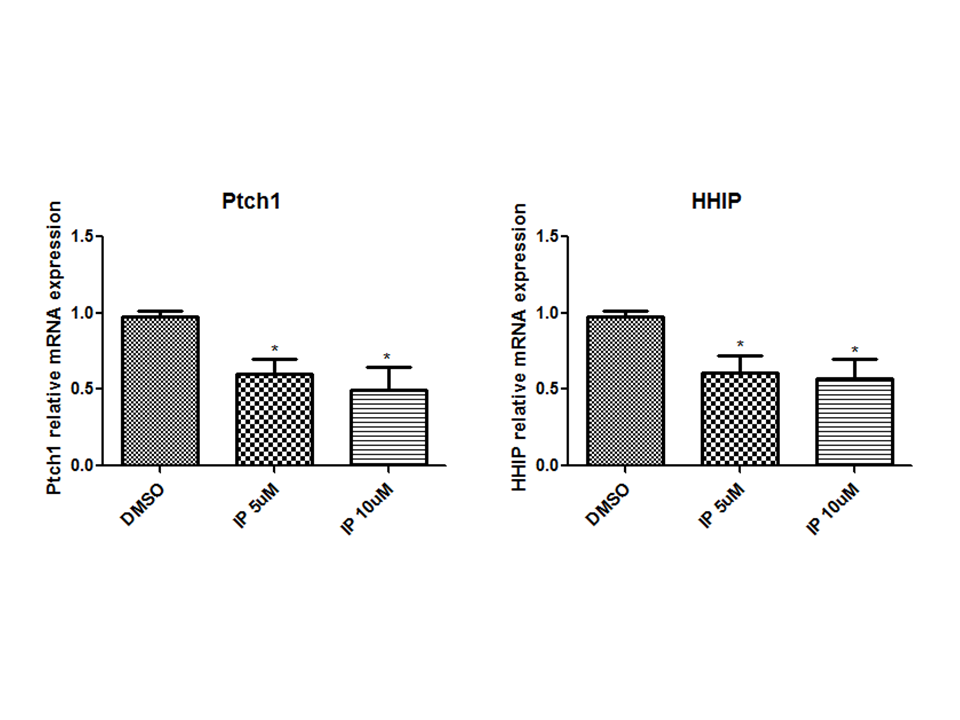

Supplement: Supplementary file 3 — Real-time PCR results showed reduced mRNA expression of key genes in the Ihh pathway, Ptch1, and Hhip, at 48 h after 5 μM and 10 μM groups. Values are the mean ± SEM. n = 3, *P < 0.05 versus the DMSO group. (TIF 161 kb) [file 13075_2019_1895_MOESM3_ESM.tif]
